# Simultaneous Patent Blue Dye Injections Aid in the Preoperative CT-Guided Localization of Multiple Pulmonary Nodules

**DOI:** 10.3390/medicina58030405

**Published:** 2022-03-09

**Authors:** Ya-Che Chen, Tsai-Wang Huang, Hsian-He Hsu, Wei-Chou Chang, Kai-Hsiung Ko

**Affiliations:** 1 Department of Radiology, Tri-Service General Hospital, National Defense Medical Center, Taipei 114, Taiwan; yachejack@gmail.com (Y.-C.C.); hsianhe@yahoo.com.tw (H.-H.H.); weichou.chang@gmail.com (W.-C.C.); 2 Division of Thoracic Surgery, Department of Surgery, Tri-Service General Hospital, National Defense Medical Center, Taipei 114, Taiwan; chi-wang@yahoo.com.tw

**Keywords:** simultaneous localization, patent blue dye, pulmonary nodule, video-assisted thoracoscopic surgery, adenocarcinoma

## Abstract

*Background and Objectives*: Clinically, a major challenge of multiple nodule localization is puncture-related pneumothorax, which may hamper the successful localization. This study aims to investigate and compare the efficacy and safety of the simultaneous and sequential patent blue dye (PBD) injections for identifying multiple pulmonary nodules during preoperative CT-guided localization. *Materials and Methods*: Sixty-one consecutive patients with multiple pulmonary nodules who underwent preoperative CT-guided localization with PBD injections between January 2020 and December 2020 were retrospectively enrolled. Of these patients, 31 patients with 64 nodules who underwent simultaneous injections were designated as the simultaneous group; the remaining 30 patients with 63 nodules who underwent sequential punctures were designated as the sequential group. The clinical and radiological features, technical information, pathological results, and procedure-related variables and complications of the two groups were reviewed and analyzed. *Results*: The localization success rate of the simultaneous group was higher than that of the sequential group (100% [64/64] vs. 93.7% [59/63], *p* = 0.041). The incidences of pneumothorax (32.3 vs. 33.3%, *p* = 0.929) and pulmonary hemorrhage (6.3 vs. 3.0%, *p* = 1) were not significantly different between the two groups, and all cases were minor, which did not require further intervention. Additionally, a significantly lower radiation dose (2.7 vs. 3.5 mSv, *p* = 0.001) and a shorter procedure time (20.95 vs. 25.28 min, *p* = 0.001) were observed in the simultaneous group than in the sequential group. *Conclusions*: Compared with the sequential method, simultaneous PBD injections may improve the localization success rate with a shorter procedure time and less radiation exposure if the patient with multiple pulmonary nodules can be approached in a single position. Further prospective studies are needed to validate these results.

## 1. Introduction

Lung cancer is the leading cause of cancer death globally [1]. Low-dose computed tomography (LDCT) screening helps in diagnosing lung cancer earlier and leads to a decrease in mortality of these patients, so it is recommended for high-risk individuals [2,3]. However, although multiple nodules may be detected during screening, the probability of having lung cancer does not necessarily correlate with the number of nodules, and each nodule should be assessed individually [4]. Currently, the mainstream method for the diagnosis and treatment of pulmonary nodules is video-assisted thoracoscopic surgery (VATS) [5]. However, the intraoperative identification of nodules is difficult when the nodules are deep-seated (>1 cm from the visceral pleura), small (≤1 cm), or are subsolid and predominantly present as ground-glass opacities on CT imaging [6].

Preoperative CT-guided nodule localization is a widely adopted approach to aid in the VATS resection for lung nodules and has been shown to have a high success rate and few complications [7,8,9]. With the increase in the number of multiple nodules detected, multiple nodule localization is being performed more frequently [10,11,12,13,14]. In our institute, the patent blue dye (PBD) injections we adopted are clinically useful for pulmonary nodule localization [8,15] and may be applied to multiple nodule localization due to the simplicity of performing them promptly. However, in clinical practice, a major challenge of multiple nodule localization is puncture-related pneumothorax; when multiple nodules are localized individually and sequentially, the use of repeated procedures poses a high risk of pneumothorax [8,11], where the collapsed lung deforms with loss of tension, disrupting the designed insertion routes. As a result, the ipsilateral pulmonary nodules may not be successfully and precisely localized. The use of simultaneous punctures may be a potential solution to overcome this issue. Although simultaneous localization of multiple nodules has been previously reported [10,11,12,16], the definitions and details of the simultaneous technique differ among reports. Furthermore, to date, simultaneous punctures with CT-guided PBD injection has not been evaluated or compared with the conventional sequential puncture method. Therefore, the aim of the study was to compare the efficacy and safety of the simultaneous PBD injections with those of the sequential approach for patients with multiple pulmonary nodules.

## 2. Materials and Methods

### 2.1. Study Population

We identified eligible patients with multiple indeterminate pulmonary nodules who underwent preoperative CT-guided localization with PBD followed by VATS in our hospital from January 2020 to December 2020. The local institutional review board and the ethics committee approved the data collection protocol, and the requirement for informed consent was waived.

Surgical indications included (1) nodules with interval growth, (2) nodules with increasing solid components, or (3) indeterminate nodules as identified by multidisciplinary discussion. Preoperative CT-guided localization was indicated for (1) nodules smaller than 3 cm, (2) nodules not adjacent to the visceral pleura, and (3) nodules that could not be easily located during the operation as agreed upon by the surgeons and radiologists. Surgeries for stable subsolid nodules with a maximal diameter or solid component <5 mm were performed at the patient’s request. All images, including CT scans obtained before, during, and after the procedure, were reviewed simultaneously by two chest radiologists (H.H.H. and K.H.K., with 30 years and 15 years of experience, respectively) in consensus. Any discrepancy in interpretation was resolved by discussion until a final consensus was reached. The simultaneous group was defined as patients with multiple nodules that underwent simultaneous needle puncture and PBD injection during one breath-hold. The sequential group was defined as patients with multiple nodules that underwent single localization punctures and sequential PBD injection. The determination of whether simultaneous or sequential localization was adopted for multiple nodule localization was based on the operator’s judgment. Generally, when the optimal path cannot be achieved via simultaneous puncture in a single position, sequential localization can be performed by changing positions.

For each patient, age, sex, smoking history and type of surgery (i.e., wedge resection, segmentectomy or lobectomy) were recorded. The CT imaging features of each lesion were also recorded, including (1) lesion size, (2) affected pulmonary lobe, (3) nodule attenuation and (4) distance between the nodule and visceral pleura. Procedure-related details, including distance between the nodule and dye, distance between the chest wall and nodule, radiation dose, and procedure time were recorded. Procedure-related complications, including pneumothorax or pulmonary hemorrhage were also recorded.

### 2.2. CT-Guided PBD Localization

Two interventional radiologists (H.H.H. and K.H.K.) performed all the localization procedures. A CT scan [64-detector row scanner (Brilliance; Philips Medical Systems, Cleveland, OH, USA)] with the low-dose setting (110 kVp, 30 mA, 1.25 pitch and 0.8-s tube rotation) was used to confirm the location of the nodules prior to procedure. Next, when the optimal path for insertion of the puncture needle was determined, we injected 2% lidocaine into the puncture site of the chest wall and a 22-gauge Chiba needle (Cook Medical, Bloomington, IN, USA) was inserted under CT guidance [15]. In the simultaneous group, patients were placed in a position suitable for all optimal insertion routes. Each puncture needle was first placed subcutaneously until reaching the extrapleural locations. Then, quick subsequent punctures into the lung parenchyma were made by the same operator within a single breath-hold. When the needle tip was within 1 cm of the nodule, 0.1–0.2 mL PBD (patent blue V 2.5%; Guerbet, Aulnay-sous-Bois, France) was injected. For deeply situated nodules, the dye was injected into the deepest area near the nodule and along the withdrawal path up to the subpleural area (<1 cm from the pleura). In contrast, patients in the sequential group were examined using a conventional method in which each of the nodules was punctured and injected with the same volume of PBD independently. The patient was repositioned to reach nodules that were not accessible in the previous position. A postprocedure CT scan was performed to check for staining, which presented as focal ground-glass opacities near the nodules and the subpleural region. After the localization procedure, patients returned to the ward and waited to be transferred to the operating room.

The procedure time was defined as the interval between the preliminary and final CT imaging scans. The radiation dose was recorded as the dose-length product (mGy-cm) and converted to the effective dose (mSv) using a conversion factor (0.014 mSv mGy^−1^ cm^−1^) [17].

### 2.3. Success Rate and Complications

A successful localization was defined as the presence of focal ground-glass opacities adjacent to the target lesions on CT scans and interoperative visualization of the dye stain on the visceral pleura, enabling complete resection of the nodule with negative margins in pathology, which was confirmed by the absence of residual lesions on postoperative CT scans. In contrast, technical failure was determined as the absence of staining with dye or the inability to clearly identify the dye stain intraoperatively. Regarding complications, a pneumothorax was recorded as asymptomatic or symptomatic based on whether respiratory symptoms such as chest pain, dyspnea, or oxygen desaturation were present and whether further intervention was required. Pulmonary hemorrhage was graded as follows according to a previously reported system [18]: 0, none; 1, less than or equal to 2 cm around the needle; 2, more than 2 cm and sublobar; 3, at least lobar; and 4, hemothorax.

### 2.4. VATS after Localization

VATS was performed for all patients on the same day after CT-guided preoperative localization. Wedge resection was performed after visualization of the dyed area in the visceral pleura via thoracoscopy. The resected specimen was then sent for frozen sectioning. The surgery was terminated if the pathology suggested benignity and if the patients had a limited cardiopulmonary reserve and nodules smaller than 2 cm with a predominant ground-glass opacity presentation [19]. Otherwise, when malignancy was confirmed, anatomic resection and mediastinal lymph node dissection was performed.

### 2.5. Statistical Analysis

Patient demographics and nodule characteristics are expressed using descriptive statistics (median with interquartile range for continuous variables and proportions for categorical variables). The chi-square test and the Mann–Whitney test were applied to compare discrete and continuous variables, respectively, between the two groups. Univariate logistic regression was used to identify risk factors for procedure-related pneumothorax. A two-sided *p* value less than 0.05 was considered statistically significant. SPSS (IBM SPSS Statistics, Version 22.0, Chicago, IL, USA) was used for statistical analysis.

## 3. Results

### 3.1. Patient and Nodule Characteristics

**Figure 1:**
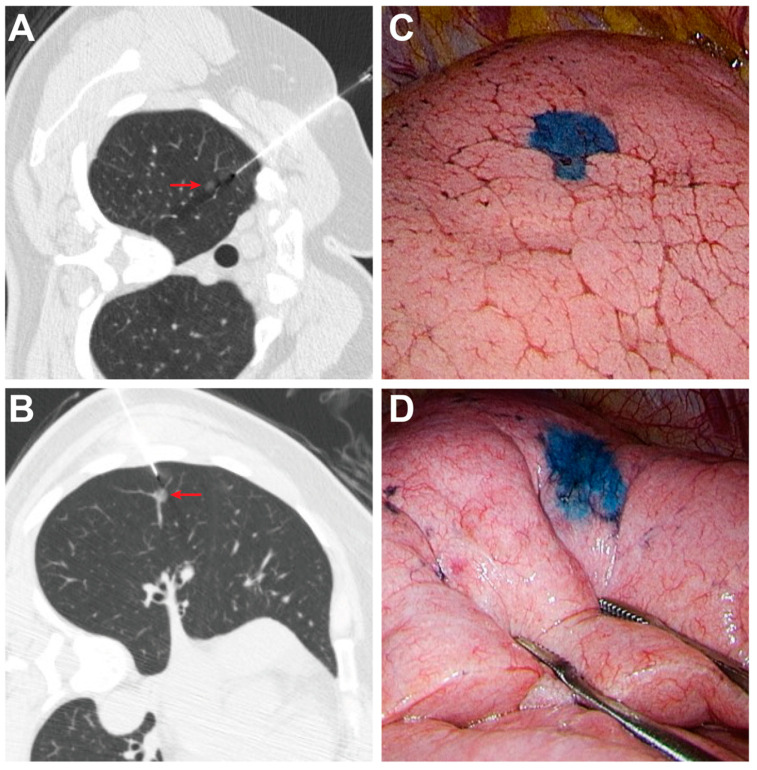
(**A**,**B**) Localization via simultaneous puncture with patent blue dye (PBD) injection of two subsolid nodules (arrows) was performed in a 58-year-old female in the lateral decubitus position; (**C**,**D**) The stained areas on the visceral pleura were obviously identified during video-assisted thoracoscopic surgery (VATS). These two nodules were proved to be invasive adenocarcinomas.

**Figure 2:**
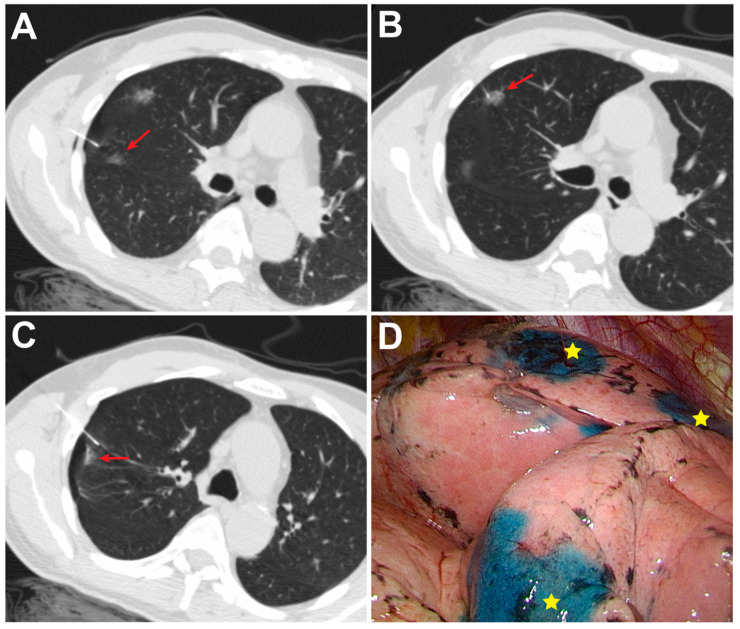
Simultaneous puncture with PBD injection of three nodules in a 70-year-old male. (**A**–**C**) Axial CT scan showed 22-gauge Chiba needles simultaneously puncturing two subsolid nodules (arrow) in the RUL and one subsolid nodule (arrow) in the RML during one breath-hold; (**D**) VATS identified PBD stains (★) of the three nodules on the visceral pleura of the RUL and RML. All pathological results indicated invasive adenocarcinoma. Abbreviations: RUL, right upper lobe; RML, right middle lobe.

The characteristics of the 61 enrolled patients and their combined 127 pulmonary nodules are shown in Table 1. In the simultaneous group, 29 patients had two nodules (Figure 1) and two patients had three nodules (Figure 2), while in the sequential group, 27 patients had two nodules and three patients had three nodules. There was no significant difference in nodule size, depth, location or attenuation of nodules between groups. The median nodule size was 7.0 mm in the simultaneous group and 6.0 mm in the sequential group. The distance between the nodule and pleural surface was 7.8 mm in the simultaneous group and 5.0 mm in the sequential group. The majority of nodules in both groups appeared as ground-glass opacities (78.1% vs. 88.9%, *p* = 0.164).

### 3.2. Preoperative Results and Complications Related to CT-Guided Localization

The preoperative results and complications related to CT-guided localization are shown in Table 2. The localization success rate in the simultaneous group was higher than that in the sequential group (100% [64/64] vs. 93.7% [59/63], *p* = 0.041). However, in the sequential group, the individual nodules of four patients were not localized due to pneumothorax. In one of these patients, the nodule became undetectable on CT due to ipsilateral lung collapse, masking the nodule location. Another two patients could not perform the second breath-hold well after pneumothorax. In the fourth patient, the puncture needle failed to reach the periphery of the nodule due to the presence of distorted anatomy. For these patients, sublobar anatomic resection was performed. The median total radiation dose and procedure time differed significantly between the two groups (Table 2), with a lower median total radiation dose (2.7 vs. 3.5 mSv, *p* = 0.001) and shorter CT-guided localization procedure time in the simultaneous group (20.95 vs. 25.28 min, *p* = 0.001). There was no interoperator difference, with the two operators demonstrating shorter procedure times in the simultaneous group than in the sequential group (20.02 vs. 24.42 min, *p* = 0.006 and 22.98 vs. 25.75 min, *p* = 0.019). More cases were performed under lateral decubitus positions in the simultaneous group than the sequential group (12 [18.8%] vs. two [3.2%], *p* = 0.018). There was no significant difference in the distance between the dye and nodule, the distance between the chest wall and nodule, or incidence of procedure-related complications (including pneumothorax and pulmonary hemorrhage) between groups. No specific risk factors associated with procedure-related pneumothorax were identified by univariate logistic regression analysis (Table 3).

### 3.3. Operative and Pathological Results

Most of the nodules in the two groups (81.3% in the simultaneous group and 88.9% in the sequential group) were treated with wedge resection (Table 4). The median operation time was equivalent between the two groups (57 vs. 54 min, *p* = 0.843). The majority of nodules (85%, 108/127) were malignancies, including invasive adenocarcinoma, minimally invasive adenocarcinoma (MIA), adenocarcinoma in situ (AIS) and metastasis.

## 4. Discussion

In this retrospective cohort, we aimed to compare the efficacy and safety of simultaneous PBD injections with sequential PBD injections in the preoperative CT-guided localization of multiple pulmonary nodules. Compared with sequential injections, simultaneous PBD injections led to a higher localization success rate (100% vs. 93.7%), a shorter procedure time (20.95 vs. 25.28 min), and a lower radiation dose (2.7 vs. 3.5 mSv). Based on our results, the use of preoperative CT-guided simultaneous PBD injections is feasible and safe for localizing multiple pulmonary nodules.

Prior studies have shown that preoperative multiple nodule localization yields a similar success rate to single nodule localization [10,11,12,20]. However, the temporal relation of each puncture in multiple nodule localization procedures (i.e., simultaneous or sequential) was not emphasized. In some studies, simultaneous localization was described as multiple localizations in the same period of the procedure [10,11,13,20,21], while in others, it was referred to as simultaneous “puncture” [12,16]. As a result, different puncture techniques may lead to different results. In our study, simultaneous localization was defined as all needle punctures performed in one breath-hold at the same time. With the use of this method, the localization success rate of our simultaneous group was among the highest reported by prior studies on multiple nodule localization (91.4–100%) [10,11,12,13,20,21]. This result reflected that the advantage of the simultaneous puncture technique—the potential to avoid the interference of the pneumothorax which further disrupted subsequent localization—might have contributed to the high success rate. Furthermore, the median procedure time in the simultaneous group was 20.95 min, shorter than the range of 24 to 56 min in prior studies [10,11,12,13,20]. There are a number of possible reasons for the shorter procedure time. First, dye injection is easier than the placement of metallic devices such as coils, which may be technically demanding [22]. Second, our patients did not change positions in the simultaneous method. Finally, the simultaneous method required fewer CT scans: when we performed simultaneous puncture, the multiple needle trajectories were designed via a preliminary CT scan with the patient remaining in the same position, whereas in the sequential method, multiple scans were needed to confirm the dye in the first nodule and locate the next. Thus, simultaneous punctures decreased the number of CT scans and reduced the time lag between scans, explaining why the entire procedure became shorter. According to our results, a simultaneous approach might be useful for operators in performing the preoperative localization for multiple nodules.

Regarding complications, pneumothorax was identified as the most common finding after transpleural needle puncture procedures [23], and its incidence ranges from 9% to 54% in lung nodule biopsy [23,24]. Older age, deeper lesion location, longer time to pass the needle through the pleura, multiple puncture, passage of the needle through a fissure, and position change are associated with increased risk of pneumothorax [15,20]. In our study, the difference in the rate of pneumothorax in the simultaneous and sequential groups was statistically nonsignificant (32.3% vs. 33.3%) and was comparable to the rates between 12.9% and 55.8% in prior studies on multiple nodule localization [10,11,12,13,20,21]. In our cohort, all our patients with pneumothorax were managed conservatively without chest tube insertion. Additionally, no particular risk factors were identified for procedure-related pneumothorax in our study. Although multiple punctures inevitably increase the risk of pneumothorax, our simultaneous puncture technique may achieve concurrent nodule targeting before pneumothorax occurs. Regarding pulmonary hemorrhage, there were no differences between the two groups (6.3% vs. 3.0%, *p* = 1), and all cases were classified as grade one.

Recently, with the advent of hybrid operating rooms (OR), intraoperative CT-guided localization has become optional [25,26,27,28]; nevertheless, despite the advantage of its one-step treatment process, several concerns remain. First, CT-guided localization requires a longer general anesthesia time, and the patient may become prone to pneumothorax risk after needle puncture due to a positive airway pressure [28]. Second, specific equipment such as fluoroscopes and C-arm cone-beam CT devices are required. The use of fluoroscopy may pose a risk of radiation exposure to the operators [16,27,29], and cone-beam CT in the hybrid OR may not provide the same image quality as multidetector CT. Therefore, the choice between intraoperative and preoperative CT-guided localization remains debated. In terms of the radiation dose, Chao et al. [16]. demonstrated that intraoperative simultaneous CT-guided localization delivered less than preoperative sequential CT-guided localization (3.05 vs. 18.65 mSv, *p* < 0.001). However, the median radiation dose in our preoperative CT-guided simultaneous group was far lower (2.7 vs. 18.65 mSv) than that in their preoperative CT-guided group and slightly lower (2.7 vs. 3.05 mSv) than that in their intraoperative group. There are several possible reasons for these discrepancies. First, they used either hookwires or dye depending on the depth of the nodule. Second, the preoperative CT-guided procedure of our study was performed under a low-dose setting. Finally, the nodules were punctured in a sequential manner in their preoperative CT-guided group and simultaneously in their intraoperative group, as in our preoperative CT-guided simultaneous group. It can therefore be considered that the different radiation doses are attributed to the different puncture techniques, with the simultaneous puncture technique yielding lower radiation exposure. We believe that simultaneous puncture with PBD injection can attain lower radiation exposure by means of highly accessible multidetector CT devices and Chiba needles in the conventional CT room with no radiation exposure to the operators.

Various localization methods and materials have been reported, including methylene blue dye [12,30], radiotracer [31], hookwires [20], and microcoils [10,11,13,21,32,33]. Of them, hookwires and microcoils are commonly used but require the operator’s expertise to minimize the risk of dislodgement and avoid increased radiation exposure when launching the metallic device [10,20,32,33]. The drawback of PBD is the diffusion effect, which was previously reported [12]. However, based on our previous experience, the injection dose may be the key point, and a lower dose (0.07 mL) of PBD injection for subpleural nodules (<1 cm from the pleura) is sufficient for good staining of the visceral pleura [15]. In clinical practice, one advantage of PBD is its simplicity, which yields a faster learning curve than the placement of hookwires or coils. Moreover, because our approach only requires a puncture needle and PBD, another advantage of PBD injections is relatively low cost when compared with hookwire or microcoil implantation and other complicated localization techniques.

There are some limitations to this study. First, due to its retrospective nature, the presence of the patient selection bias is inevitable. Second, this study included a relatively small population in a single center, and a larger cohort may be required to clarify these findings and their reproducibility. Third, the results were not compared to those of other localization modalities and tools. Future studies focused on different methods may be needed. Nonetheless, based on our results, the simultaneous PBD injections may achieve a better success rate with lower radiation exposure and minor complications, indicating their clinical feasibility and safety.

## 5. Conclusions

In this paper, we demonstrated that if the patient with multiple nodules can be approached in a single position, the use of simultaneous PBD injections may have a high success rate, a short procedure time, and low radiation exposure in performing preoperative CT-guided localization, suggesting that it may be an effective and safe localization technique that can facilitate the precise resection via the VATS procedure. However, further larger and prospective studies are needed for validation of the current results. 

## Figures and Tables

**Table 1:** Nodule and patient characteristics.

Variables	Simultaneous (*n* = 31)	Sequential (*n* = 30)	*p* Value
Number of patients with			
two nodules, *n*	29	27	
three nodules, *n*	2	3	
Age, years			
Median (IQR)	65 (58–70)	61 (50–67)	0.103
Range	[38–83]	[29–81]	
Sex, *n* (%)			0.939
Male	8 (25.8)	8 (26.7)	
Female	23 (74.2)	22 (73.3)	
Smoking status, *n* (%)			0.955
Never	26 (83.9)	25 (83.3)	
Current or former	5 (16.1)	5 (16.7)	
Size, mm			
Median (IQR)	7 (5–9)	6 (4.5–8)	0.058
Range	[3–25]	[2–18]	
Depth, mm			
Median (IQR)	7.8 (3–14.3)	5 (1.6–12.8)	0.21
Range	[0–34]	[0–33]	
Pulmonary lobe, *n* (%)			0.132
RUL	21 (32.8)	24 (38.1)	
RML	2 (3.1)	8 (12.7)	
RLL	16 (25)	15 (23.8)	
LUL	14 (21.9)	6 (9.5)	
LLL	11 (17.2)	10 (15.9)	
Nodule attenuation, *n* (%)			0.164
Ground-glass opacity	50 (78.1)	56 (88.9)	
Part solid	2 (3.1)	3 (4.8)	
Solid	10 (15.6)	4 (6.3)	
Cavitation	2 (3.1)	0 (0)	

Abbreviations: RUL, right upper lobe; RML, right middle lobe; RLL, right lower lobe; LUL, left upper lobe; LLL, left lower lobe; IQR, interquartile range.

**Table 2:** Preoperative results and complications of CT-guided localization.

Variable	Simultaneous (*n* = 31)	Sequential (*n* = 30)	*p* Value
Localization success, %	100	93.7	0.041
Effective dose, mSv	2.7 (2.43–3.19)	3.5 (2.77–3.89)	0.001
Procedure time, min	20.95 (16.45–22.47)	25.28 (22.85–27.16)	0.001
Operator 1, min (*n* = 27)	20.02 (17.19–24.32)	24.42 (21.42–26.47)	0.006
Operator 2, min (*n* = 34)	22.98 (18.82–24.98)	25.75 (23.82–28.88)	0.019
Distance between the nodule and the dye, mm	0.5 (0–4.9)	3 (0–10)	0.058
Distance between the chest wall and the nodule, mm	47.5 (38–65.75)	55 (42–66)	0.229
Position, *n* (%)			0.018
Supine	27 (42.2)	29 (46.0)	
Prone	25 (39.1)	32 (50.8)	
Lateral decubitus	12 (18.8)	2 (3.2)	
Pneumothorax, *n* (%)	10 (32.3)	10 (33.3)	0.929
Pulmonary hemorrhage, *n* (%)	2 (6.3)	1 (3.0)	1

**Table 3:** Risk factors for procedure-related pneumothorax.

Variable	*p* Value	OR (95% CI)
Age, years	0.858	1.004 (0.959–1.052)
Sex (ref: F)	0.093	2.750 (0.843–8.967)
Smoking status	0.596	1.458 (0.361–5.894)
Size, mm	0.532	1.372 (0.509–3.703)
Depth, mm	0.582	1.130 (0.731–1.746)
Simultaneous (ref: sequential)	0.929	0.952 (0.327–2.774)
Operator (ref: operator 1)	0.640	1.295 (0.438–3.834)
Pulmonary lobe (ref: RUL)		
RML	0.327	2.000 (0.500–7.997)
RLL	0.922	0.952 (0.359–2.526)
LUL	0.791	0.857 (0.274–2.679)
LLL	0.706	1.231 (0.419–3.613)
Attenuation (ref: GGO)		
Part solid	0.885	1.145 (0.183–7.156)
Solid	0.056	0.132 (0.017–1.049)
Cavitation	0.999	-
Position (ref: supine)		
Prone	0.395	0.717 (0.334–1.543)
Decubitus	0.078	0.239 (0.049–1.171)

Abbreviations: RUL, right upper lobe; RML, right middle lobe; RLL, right lower lobe; LUL, left upper lobe; LLL, left lower lobe; GGO, ground-glass opacity.

**Table 4:** Operative and pathological results.

Variable	Simultaneous (*n* = 31)	Sequential (*n* = 30)	*p* Value
Surgical procedure, *n* (%)			0.077
Wedge resection	52 (81.3)	56 (88.9)	
Segmentectomy	7 (10.9)	7 (11.1)	
Lobectomy	5 (7.8)	0 (0)	
Operation time, min			
Median (IQR)	57 (42–106)	54 (42.5–99.75)	0.834
Range	[27–357]	[24–175]	
Inpatient days, days			
Median (IQR)	7 (5–9)	7 (5–8)	0.775
Range	[4–18]	[4–17]	
Pathology, *n* (%)			0.077
Invasive adenocarcinoma	33 (51.6)	35 (55.6)	
MIA	13 (20.3)	9 (14.3)	
AIS	4 (6.3)	8 (12.7)	
Metastasis	6 (9.4)	0 (0)	
AAH	0 (0)	3 (4.8)	
Carcinoid tumor	1 (1.6)	0 (0)	
Focal interstitial fibrosis	4 (6.3)	5 (8)	
Chronic granulomatous inflammation	0 (0)	1 (1.6)	
Hemorrhage with congestion	1 (1.6)	2 (3.2)	
Minute pulmonary meningothelial-like nodule	2 (3.1)	0 (0)	

Abbreviations: AAH, atypical adenomatous hyperplasia; AIS, adenocarcinoma in situ; MIA, minimally invasive adenocarcinoma; IQR, interquartile range.

## Data Availability

The raw data supporting the conclusions of this article will be made available by the authors, without undue reservation.

## References

[1] Sung H., Ferlay J., Siegel R.L., Laversanne M., Soerjomataram I., Jemal A., Bray F. (2021). Global cancer statistics 2020: GLOBOCAN estimates of incidence and mortality worldwide for 36 cancers in 185 countries. CA Cancer J. Clin..

[2] Smith R.A., Andrews K.S., Brooks D., Fedewa S.A., Manassaram-Baptiste D., Saslow D., Brawley O.W., Wender R.C. (2017). Cancer screening in the United States, 2017: A review of current American Cancer Society guidelines and current issues in cancer screening. CA Cancer J. Clin..

[3] De Koning H.J., van der Aalst C.M., de Jong P.A., Scholten E.T., Nackaerts K., Heuvelmans M.A., Lammers J.J., Weenink C., Yousaf-Khan U., Horeweg N. (2020). Reduced lung-cancer mortality with volume CT screening in a randomized trial. N. Engl. J. Med..

[4] Heuvelmans M.A., Walter J.E., Peters R.B., Bock G.H., Yousaf-Khan U., Aalst C.M.V., Groen H.J.M., Nackaerts K., Ooijen P.M.V., Koning H.J. (2017). Relationship between nodule count and lung cancer probability in baseline CT lung cancer screening: The NELSON study. Lung Cancer.

[5] Congregado M., Merchan R.J., Gallardo G., Ayarra J., Loscertales J. (2008). Video-assisted thoracic surgery (VATS) lobectomy: 13 years’ experience. Surg. Endosc..

[6] Suzuki K., Nagai K., Yoshida J., Ohmatsu H., Takahashi K., Nishimura M., Nishiwaki Y. (1999). Video-assisted thoracoscopic surgery for small indeterminate pulmonary nodules: Indications for preoperative marking. Chest.

[7] Chen J.R., Tseng Y.H., Lin M.W., Chen H.M., Chen Y.C., Chen M.C., Lee Y.F., Chen J.S., Chang Y.C. (2019). Safety and efficacy of computed tomography-guided dye localization using patent blue V for single lung nodule for video-assisted thoracoscopic surgery: A retrospective study. Ann. Transl. Med..

[8] Lin M.W., Tseng Y.H., Lee Y.F., Hsieh M.S., Ko W.C., Chen J.Y., Hsu H.H., Chang Y.C., Chen J.S. (2016). Computed tomography-guided patent blue vital dye localization of pulmonary nodules in uniportal thoracoscopy. J. Thorac. Cardiovasc. Surg..

[9] McDermott S., Fintelmann F.J., Bierhals A.J., Silin D.D., Price M.C., Ott H.C., Shepard J.O., Mayo J.R., Sharma A. (2019). Image-guided preoperative localization of pulmonary nodules for video-assisted and robotically assisted surgery. Radiographics.

[10] Xu Y., Ma L., Sun H., Huang Z., Zhang Z., Xiao F., Ma Q., Lin J., Xie S. (2021). The utility of simultaneous CT-guided localization for multiple pulmonary nodules using microcoil before video-assisted thoracic surgery. BMC Pulm. Med..

[11] Hu L., Gao J., Hong N., Liu H., Chen C., Zhi X., Sui X. (2021). Simultaneous preoperative computed tomography-guided microcoil localizations of multiple pulmonary nodules. Eur. Radiol..

[12] Lin C.Y., Chang C.C., Huang L.T., Chung T.J., Liu Y.S., Yen Y.T., Tseng Y.L. (2021). Computed tomography-guided methylene blue localization: Single vs. multiple lung nodules. Front. Med..

[13] Teng F., Wu A.L., Yang S., Lin J., Xian Y.T., Fu Y.F. (2020). Preoperative computed tomography-guided coil localization for multiple lung nodules. Ther. Adv. Respir. Dis..

[14] Tseng Y.H., Lee Y.F., Hsieh M.S., Chien N., Ko W.C., Chen J.Y., Lee J.M., Huang P.M., Lin M.W., Chen J.S. (2016). Preoperative computed tomography-guided dye injection to localize multiple lung nodules for video-assisted thoracoscopic surgery. J. Thorac. Dis..

[15] Ko K.H., Huang T.W., Lee S.C., Chang W.C., Gao H.W., Hsu H.H. (2019). A simple and efficient method to perform preoperative pulmonary nodule localization: CT-guided patent blue dye injection. Clin. Imaging.

[16] Chao Y.K., Fang H.Y., Pan K.T., Wen C.T., Hsieh M.J. (2020). Preoperative versus intraoperative image-guided localization of multiple ipsilateral lung nodules. Eur. J. Cardiothorac. Surg..

[17] McCollough C., Edyvean S., Geise R., Gould B., Keat N., Huda W., Judy P., Kalender W., McNitt-Gray M., Morin R. (2008). The Measurement, Reporting, and Management of Radiation Dose in CT.

[18] Tai R., Dunne R.M., Trotman-Dickenson B., Jacobson F.L., Madan R., Kumamaru K.K., Hunsaker A.R. (2016). Frequency and severity of pulmonary hemorrhage in patients undergoing percutaneous CT-guided transthoracic lung biopsy: Single-institution experience of 1175 cases. Radiology.

[19] Kato H., Oizumi H., Suzuki J., Hamada A., Watarai H., Nakahashi K., Sadahiro M. (2017). Thoracoscopic wedge resection and segmentectomy for small-sized pulmonary nodules. J. Vis. Surg..

[20] Zhong Y., Xu X.Q., Pan X.L., Zhang W., Xu H., Yuan M., Kong L.Y., Pu X.H., Chen L., Yu T.F. (2017). Retrospective evaluation of safety, efficacy and risk factors for pneumothorax in simultaneous localizations of multiple pulmonary nodules using hook wire system. Cardiovasc. Interv. Radiol..

[21] Fu Y.F., Gao Y.G., Zhang M., Wang T., Shi Y.B., Huang Y.Y. (2019). Computed tomography-guided simultaneous coil localization as a bridge to one-stage surgery for multiple lung nodules: A retrospective study. J. Cardiothorac. Surg..

[22] Sun S.H., Gao J., Zeng X.M., Zhang Y.F. (2021). Computed tomography-guided localization for lung nodules: Methylene-blue versus coil localization. Minim. Invasive Ther. Allied Technol..

[23] Boskovic T., Stanic J., Pena-Karan S., Zarogoulidis P., Drevelegas K., Katsikogiannis N., Machairiotis N., Mpakas A., Tsakiridis K., Kesisis G. (2014). Pneumothorax after transthoracic needle biopsy of lung lesions under CT guidance. J. Thorac. Dis..

[24] Dennie C.J., Matzinger F.R., Marriner J.R., Maziak D.E. (2001). Transthoracic needle biopsy of the lung: Results of early discharge in 506 outpatients. Radiology.

[25] Lempel J.K., Raymond D.P. (2019). Intraoperative percutaneous microcoil localization of small peripheral pulmonary nodules using cone-beam CT in a hybrid operating room. AJR Am. J. Roentgenol..

[26] Yang S.M., Ko W.C., Lin M.W., Hsu H.H., Chan C.Y., Wu I.H., Chang Y.C., Chen J.S. (2016). Image-guided thoracoscopic surgery with dye localization in a hybrid operating room. J. Thorac. Dis..

[27] Chao Y.K., Pan K.T., Wen C.T., Fang H.Y., Hsieh M.J. (2018). A comparison of efficacy and safety of preoperative versus intraoperative computed tomography-guided thoracoscopic lung resection. J. Thorac. Cardiovasc. Surg..

[28] Chen P.H., Hsu H.H., Yang S.M., Tsai T.M., Tsou K.C., Liao H.C., Lin M.W., Chen J.S. (2018). Preoperative dye localization for thoracoscopic lung surgery: Hybrid versus computed tomography room. Ann. Thorac. Surg..

[29] Hsieh M.J., Fang H.Y., Lin C.C., Wen C.T., Chen H.W., Chao Y.K. (2018). Single-stage localization and removal of small lung nodules through image-guided video-assisted thoracoscopic surgery. Eur. J. Cardiothorac. Surg..

[30] Lenglinger F.X., Schwarz C.D., Artmann W. (1994). Localization of pulmonary nodules before thoracoscopic surgery: Value of percutaneous staining with methylene blue. AJR Am. J. Roentgenol..

[31] Starnes S.L., Wolujewicz M., Guitron J., Williams V., Scheler J., Ristagno R. (2018). Radiotracer localization of nonpalpable pulmonary nodules: A single-center experience. J. Thorac. Cardiovasc. Surg..

[32] Lizza N., Eucher P., Haxhe J.P., De Wispelaere J.F., Johnson P.M., Delaunois L. (2001). Thoracoscopic resection of pulmonary nodules after computed tomographic-guided coil labeling. Ann. Thorac. Surg..

[33] Liu L., Zhang L.J., Chen B., Cao J.M., Lu G.M., Yuan L., Li K., Xu J. (2014). Novel CT-guided coil localization of peripheral pulmonary nodules prior to video-assisted thoracoscopic surgery: A pilot study. Acta Radiol..

